# Genomics as part of Portuguese undergraduate nursing programs: are we moving in the right direction?

**DOI:** 10.1007/s12687-025-00787-2

**Published:** 2025-03-26

**Authors:** Maria João Silva, Maria do Céu Barbieri-Figueiredo, Marcia Van Riper, Milena Paneque

**Affiliations:** 1 ICBAS – School of Medicine and Biomedical Sciences of University of Porto, Porto, Portugal; 2 University of North Carolina, Chapel Hill, USA; 3 CGPP – Centre for Predictive and Preventive Genetics, University of Porto, Porto, Portugal; 4 IBMC – Institute of Molecular and Cellular Biology, University of Porto, Porto, Portugal; 5 i3S – Institute for Research and Innovation in Health, University of Porto, Porto, Portugal

**Keywords:** Genomics, Nursing education, Curriculum, Undergraduate, Content analysis, Portugal

## Abstract

The integration of genomics into nursing education has been a growing focus in recent years, as the role of genomics in healthcare continues to expand. Although the fundamental role of nurses in integrating genomic information into patient care is well-documented in the literature, studies have consistently highlighted significant gaps in nurses’ understanding of these topics, impacting their ability to provide comprehensive care. This study aims to explore how genomic knowledge is taught in Portuguese undergraduate nursing education at a national level. A deductive content analysis was performed on explicit genomic content in each course specification within the nursing programs. A total of 478 course descriptions from 12 nursing programs were analyzed. Of these, only 25 courses (5.2%) explicitly referenced genomic content. Results reveal significant variability across programs, with some covering a considerable number of genetic topics, while others showed minimal or no coverage of genomic topics. Results also show that topics related to basic molecular biology and fundamental genetic principles tend to be more emphasized in nursing curricula compared to those focused on psychosocial aspects or patient-centered care. This inconsistency highlights the lack of a standardized approach to integrating genomics into nursing education. These findings suggest that the current approach to Portuguese nursing education is insufficient for preparing nurses to effectively address genomic issues in patient care and research. This research argues for a more systematic, early, and consistent integration of genomics across all nursing programs to ensure that future nurses are well-equipped to meet the challenges of modern healthcare, ultimately improving patient outcomes.

## Background

The Human Genome Project, completed in 2003, marked the beginning of the “genomic era” transforming healthcare by enabling rapid genetic diagnosis and the development of tailored therapies that improve patient outcomes (Thomas et al. 2023). According to WHO (2024) genomics is the study of the complete set of genes, the way genes work, interact with each other and with the environment. These include both well-known single-gene disorders, and complex multifactorial diseases (WHO 2024). Genomic healthcare applications cover the entire lifespan, from prenatal testing and newborn screenings to predicting the risk of common health conditions like cancer and cardiovascular diseases, as well as informing pharmacogenomic decisions, ultimately enhancing personalized healthcare outcomes (Calzone et al. 2024). The continued progress of genomics is opening new doors for precision healthcare that considers individual lifestyle, genetics, behaviors, and environment context and facilitates interventions aimed at helping individuals achieve well-being and optimal health (Hulick and Ilbawi 2024). Furthermore, with lower costs, more rapid sequencing technologies and the remarkable growth and acceptance of artificial intelligence, genomic healthcare has become increasingly mainstream, offering more effective diagnostic and treatment options (Costa et al. 2024; Johnson et al. 2021; Wang et al. 2023).

### The role of nurses in genomics and precision healthcare

In the context of precision healthcare, multidisciplinary collaboration is essential for effective patient diagnosis and treatment (Zhu et al. 2024). As the largest workforce in healthcare, nurses are uniquely positioned to lead the integration of emerging genomic information into patient, family, and community care (Dewell et al. 2024). With their central and continuous role in patient centered-care, nurses are often the first point of contact for patients and play a critical role in genetic-related healthcare, supporting patients as they navigate the complexities of genetic testing and personalized health information (Clary-Muronda and Smith 2024; Dewell et al. 2020; Tonkin et al. 2020).

The influence of genomics on nursing practice is expanding across various roles and competencies. Nurses should engage in essential activities such as risk assessment, family history collection, education, and advocacy for individuals and groups who could benefit from genetic services (Calzone et al. 2018a, b). By actively identifying individuals at increased risk for genetic conditions and guiding them through genetic testing processes, nurses help demystify genomics for patients and families, fostering a more informed and supportive healthcare experience.

The integration of genomics into nursing practice also allows nurses to play a key role in promoting equitable access to genomics-based healthcare (Paneque et al. 2022). As advocates, nurses are instrumental in ensuring that patients understand the implications of genomic information, particularly for those who may benefit from tailored interventions based on their genomic individuality (Schluter 2023). This advocacy extends beyond individual care, as nurses work with families and communities to encourage awareness and informed decision-making regarding genetic services.

Moreover, by working closely with physicians, pharmacists, genetic counselors, and laboratory personnel, nurses contribute to the development of comprehensive and holistic care strategies that enhance patient outcomes and promote adherence to treatment regimen, ultimately facilitating the successful implementation of precision healthcare (Paneque et al. 2017; Zhu et al. 2024).

### Gaps in nursing education in the genomic era

Although the fundamental role of nurses in integrating genomic information into patient care is well-documented in the literature, studies have consistently highlighted significant gaps in nurses’ understanding of these topics, impacting their ability to provide comprehensive care. In a systematic review about the nurses’ knowledge in genetics, Skirton & Godino (2012) found that both perceived and actual knowledge of genetics among nurses is poor, and the amount of genetics education they receive is insufficient. However, nurses are open to genetics education, highlighting the need for initiatives that integrate scientific principles with practical applications to enhance their ability to utilize genetics effectively in patient care.

Studies conducted across various countries have assessed students and nurses’ baseline knowledge and attitudes in genomics. While most nurses recognize the importance of genomics for their practice, they frequently lack the knowledge, confidence, and skills necessary to effectively integrate these concepts into patient care (Adejumo et al. 2021; Chow et al. 2023; Dewell et al. 2020; Parviainen et al. 2023; Seven et al. 2015; Wang et al. 2023). This lack of knowledge not only impairs nurses’ ability to educate patients but also poses significant challenges to the effective integration of genomic care into clinical practice.

Since the early 2000s, global nursing organizations have stressed the need to incorporate genomic knowledge and skills into nursing education at all levels, from pre-licensure baccalaureate programs to advanced graduate studies (Camak 2016; Dewell et al. 2020; Sharoff 2015; Wright et al. 2018). However, there is significant variation in how and to what extent nursing programs have incorporated genomics into their curricula.

The major obstacles to the full integration of genomics into nursing education include: gaps in awareness and knowledge among nursing faculty, leading to a lack of professional involvement in genomics; insufficient recognition of its importance at the government and regulatory levels; resource limitations such as time and funding, and the shortage of qualified faculty to teach genomics; inadequate focus on patient education; and the absence of robust evidence on outcomes (Barbato et al. 2019; Calzone et al. 2018a, b; Read and Ward 2016; Smania et al. 2022).

Recently, a systematic review of genomic education for nurses revealed significant gaps, including a lack of validated tools to assess outcomes and limited focus on psychosocial and counselling skills like genetic risk assessments. While interventions improved cognitive and affective knowledge initially, retention and practical application remained inconsistent. This review underscores the need to integrate genomic competencies into nursing curricula to prepare nurses for precision health (McLaughlin et al. 2024).

Clary-Muronda and Smith (2024) implemented an interprofessional genetics course for nursing students, using active learning strategies like debates and case studies to enhance reasoning and critical thinking. Despite challenges like workload and varying prior knowledge, the course highlighted the need for consistent genomic content in curricula and innovative teaching methods to prepare nurses for precision health.

In fact, the literature states that since the review of Skirton & Godino (2012), the genomic knowledge has not improved. However, a scoping review examined the state of nursing in Omics between 2012 e 2024, found a consistent growth in the body of literature. Despite the growing number of publications, implementation of genomics in nursing practice remains limited, reflecting the delay between discovery and clinical application (Thomas et al. 2023).

Although the need to integrate genomics into nursing education remains a priority, effective leadership and clear regulatory frameworks in genomic nursing are also crucial for preparing the next generation of nurses with the necessary genomic competencies. In countries such as the United States, Japan, and the United Kingdom, essential genomics competencies for nurses have been established (Calzone et al. 2018a, b; Greco et al. 2012; Kirk et al. 2014).

Competency standards for all nurses, from general to advanced practice, can be developed or adapted to fit the local context. Existing frameworks, such as the Essentials for Genetics and Genomics Nursing from the USA, can be reviewed and adjusted for local application (Abad and Sur 2022). This will help identify gaps in competencies and guide the inclusion of genetics/genomics in nursing curricula at both the undergraduate and advanced practice levels.

Calzone et al. (2024) updated the essential genomic nursing competencies, initially established in 2005 and revised in 2009 and 2023. These competencies provide a foundational framework for all registered nurses, regardless of academic preparation, role, or specialty. The 2023 update incorporates advances in genomic technology and expanded evidence-based clinical applications, including pharmacogenomics and its integration into healthcare guidelines.

Despite these advancements, the integration of genomic competencies into nursing curricula and clinical practice remains inconsistent, underscoring the need for enhanced training and ongoing education to meet the demands of modern genomic healthcare.

### Portuguese educational context

In Portugal, the Bachelor of Science in Nursing (BSc) program spans four years, amounting to 240 ECTS (European Credit Transfer and Accumulation System) credits. The curriculum is organized into core and elective courses, distributed across eight semesters. The courses include theoretical instruction, practical training, and research components. Nursing schools have the autonomy to design their specific programs, provided they adhere to national regulations established by the Ministry of Education and competency requirements established by the nursing regulatory boards. The higher education system in Portugal encompasses university, polytechnic, non-integrated higher education, and private institutions. Nursing schools are integrated across these different sectors.

In Portugal, limited research has been conducted on the implications of advancements in genomics for nursing education. The existing studies are primarily focused on clinical practice (Reisinho et al. 2020), leaving significant gaps in understanding the knowledge deficits of Portuguese nurses regarding genomics. This highlights the urgent need for research addressing these gaps to ensure that nursing professionals are adequately equipped to meet the demands of genomic healthcare.

### Aim

The aim of this study was to explore how genomic knowledge is taught in Portuguese undergraduate nursing education at a national level.

This study was based on two main questions: (1) Do the present nursing curriculums provide nursing students with the opportunity to learn about genomics? and (2) Do Portuguese nursing programs match the established genomic topics suggested by literature?

## Methods

### Research design

The genomic content in nursing curriculum was explored using a quantitative approach of a documentary analysis. More specifically, this study utilized deductive content analysis which pertains to describe explicit genomic content in each course specification within the nursing programs. The categories were established a priori with these categories being grounded in the suggested components proposed by (Skirton et al. 2012) for nursing and midwifery curricula. The work developed by (Skirton et al. 2012) discusses the necessity of integrating genomic knowledge and skills into nursing and midwifery curricula. Through a workshop with experts, essential components for genomic education were identified for inclusion in nursing education. (Skirton et al. 2012) suggest detailed learning outcomes, specific topics to be included in curriculum and learning tools to facilitate the incorporation of genomics into nursing education. In our research the analysis focused on identifying genomic content, defined according to specific topics outlined in Skirton’s framework. The specific topics used as categories for content analysis in the nursing programs are presented in Table 1.

**Table 1:** Specific topics to be included in curriculum used as categories

▪ Mendelian inheritance patterns
▪ Description of different forms of testing and their implications
▪ Description of chromosomes, genes and DNA and how they relate to each other
▪ Description of different causes of genetic disease
▪ Transcription of DNA to protein structure
▪ Communicating genetic information to families
▪ Psychosocial impact of a genetic condition on the family
▪ Ethical issues about genetic status
▪ Sources of relevant and reliable genetic information
▪ Profile and location of local/regional/national genetic services
▪ Preventive actions or treatment
▪ Family history of a disease or condition
▪ Differences between inherited disorder and genetic susceptibility
▪ Risk assessment

### Sample and data collection

A comprehensive list of nursing programs in Portugal was obtained from the official website of the Portuguese Ministry of Education. Of the 39 available BSc nursing programs, 12 were selected for analysis using a purposive sampling technique. This involved choosing the nursing programs with the highest number of students from each of the different higher education institutions that impart undergraduate nursing education (university, polytechnic, non-integrated higher education, and private). The total number of students admitted in the academic year during which this study was conducted, across the 39 Portuguese nursing programs, is 2979. The 12 selected programs, which were chosen based on their higher enrollment numbers, account for 1502 students, representing 50,4% of the total enrollment. This ensures that the sample is highly representative of the nursing student population in Portugal. By selecting the nursing programs with the highest number of students, the analysis reflects a substantial portion of the nursing student body, making the findings more generalizable and applicable to the national context of nursing education. Additionally, the selection of programs from different regions of Portugal ensures that the sample reflects the geographical diversity of the nursing programs.

Publicly accessible documents, such as program outlines and course specifications, were collected from institutional websites between May and September of 2024. Documents included overviews of entire programs, as well as detailed course descriptions outlining programmatic contents, learning objectives, and assessment methods.

### Searching and screening

The search process involved navigating the websites of selected nursing schools to locate curriculum documents. Navigation menus and search functions were used to identify relevant files. This approach offers key advantages, including cost-effectiveness, accessibility, and non-reactivity, as the documents already exist and are not influenced by the research process. However, limitations include potential incompleteness or outdated information on institutional websites, which may impact data comprehensiveness.

### Data extraction

For each nursing program included in the study, curriculum documents were carefully reviewed to extract relevant information. The analysis focused on identifying the presence of genomic content within program overviews and course descriptions. Particular attention was paid to the course typology, specifically whether the courses were biomedical, psychosocial, or nursing-related in nature. Additionally, the alignment of course objectives with the topics outlined by Skirton et al. (2012) was examined. The documents were downloaded and imported into MAXQDA software for further analysis.

Data were manually coded to ensure that both explicit references to genetics and implicit mentions related to broader genomic education were captured. The extracted information was systematically organized to facilitate further analysis, with emphasis on ensuring consistency across all programs.

### Data analysis

Data analysis was conducted using deductive content analysis techniques, as outlined by (Elo and Kyngas 2008). Documents were read thoroughly, and data were categorized into themes aligned with Skirton’s framework. This involved coding relevant sections, organizing codes into categories, and identifying patterns. To ensure a systematic and rigorous deductive content analysis, a set of keywords was predefined for each genomic topic based on Skirton’s framework. These keywords, detailed in Table 2, were selected to capture essential aspects of each topic and served as objective indicators during the coding process. Course specifications were carefully examined, and a topic was considered covered if relevant keywords appeared in the course description in an appropriate context. The process was iterative, ensuring that all relevant content was captured. The data coding was reviewed multiple times, and discrepancies were resolved through discussion among the researchers. This structured approach minimized subjectivity and enhanced consistency in data analysis, ensuring that the identification of genomic content across nursing programs was reliable and replicable. Content analysis was conducted using the MAXQDA software, which facilitated the organization and analysis of the data. Summary statistics were used to describe the frequency of genomic content, and qualitative analysis provided context on the depth and quality of coverage.

**Table 2:** Predefined keywords for each specific topic

Specific topics of genomic content	Predefined keywords
Mendelian inheritance patterns	Mendelian inheritance, dominant, recessive, autosomal, X-linked, homozygous, heterozygous.
Description of different forms of testing and their implications	Genetic testing, screening, diagnostic testing, predictive testing, genomic panels, next-generation sequencing, carrier testing, prenatal testing, exome sequencing.
Description of chromosomes, genes and DNA and how they relate to each other	Chromosomes, genes, DNA structure, mitosis, meiosis, genetic code, gene expression, molecular genetics, alleles, nucleotides, human genome.
Description of different causes of genetic disease	Genetic disorders, mutations, gene defects, chromosomal abnormalities, inherited diseases, environmental triggers, epigenetics, teratogens, multifactorial causes (include specific genetic disorders).
Transcription of DNA to protein structure	Transcription, translation, RNA, protein synthesis, gene expression, codons, RNA polymerase, splicing, amino acids.
Communicating genetic information to families	Genetic counseling, communication, family history, genetic information, result interpretation.
Psychosocial impact of a genetic condition on the family	Psychosocial impact, family dynamics, coping, genetic condition, family support, emotional response, family support, genetic distress, identity impact, genetic stigma.
Ethical issues about genetic status	Ethics, privacy, informed consent, genetic discrimination, confidentiality, genetic ownership, autonomy, disclosing genetic information to relatives.
Sources of relevant and reliable genetic information	Genetic databases, genetic resources, genetic counseling resources, educational websites.
Profile and location of local/regional/national genetic services	Genetic services, genetic counseling services, regional genetic centers, geneticists, referral pathways.
Preventive actions or treatment	Preventive actions, treatment options, gene therapy, risk reduction, healthcare strategies. early detection, targeted therapies, monitoring programs, pharmacogenomics.
Family history of a disease or condition	Family history, genetic predisposition, hereditary conditions, family tree, pedigree, ancestral genetic risk, disease markers, family linkage.
Differences between inherited disorder and genetic susceptibility	Inherited disorder, genetic susceptibility, environmental factors, inheritance patterns, polygenic traits, risk factors, familial clustering.
Risk assessment	Risk assessment, genetic risk, probability estimates, risk stratification.

### Ethical considerations

Since all data was publicly available through institutional websites, ethical approval was not required. To maintain confidentiality, the names of the nursing programs were anonymized by assigning each program a unique code. This ensured that individual programs could not be identified in the published results. Additionally, data handling adhered to principles of integrity and accuracy, safeguarding the validity of the analysis.

## Results

A total of 478 documents containing course descriptions were retrieved, of which 412 were core and 66 were elective courses comprising 1,479 pages. By nursing program, the median number of courses was 39,8 (range from 29 to 61).

These documents represent the curricula of 12 nursing programs (NP001 to NP012), which collectively offered 478 courses. Of these, only 25 courses (5.2%) explicitly referenced genomic content, as shown in Table 3. The distribution of courses that included genomic topics varied significantly among the nursing programs. For instance, NP004 had the highest number, with five courses addressing genomics, while NP010 did not include any courses with genomic references.

**Table 3:** Genomic content in courses specifications

			Genomic content (*n* – number of codified contents from a total of 58; % of the 58)														
Nursing Programs	Total of courses (n)	Courses with genomic content (n, %)	Mendelian inheritance patterns	Description of different forms of testing and their applications	Description of chromosomes, genes and DNA and how they relate to each other	Description of different causes of genetic disease	Transcription of DNA to protein structure	Communicating genetic information to families	Psychosocial impact of a genetic condition on the family	Ethical issues about genetic status	Sources of relevant and reliable genetic information	Profile and location of local/regional/nation genetic services	Preventive actions or treatment	Family history of a disease or condition	Differences between inherited disorder and genetic susceptibility	Understanding of basic risk assessment	Genomic Content (n of 14)
NP001	36	4	1	0	1	3	2	1	0	1	0	1	0	1	0	2	9
NP002	31	1	0	0	0	0	0	0	0	2	0	0	0	0	0	0	1
NP003	43	2	0	0	1	0	0	0	0	2	0	0	0	0	0	0	2
NP004	61	5	1	0	2	2	2	1	2	2	0	0	1	0	2	0	9
NP005	30	2	0	0	2	0	0	0	0	0	0	0	0	0	0	0	1
NP006	31	2	1	1	1	1	1	0	0	0	0	0	1	0	1	0	7
NP007	50	1	0	1	0	1	0	0	0	0	0	0	0	0	0	1	3
NP008	48	2	0	0	1	2	1	0	0	0	0	0	0	0	2	0	4
NP009	39	2	0	1	0	0	1	0	0	0	0	0	1	0	0	0	3
NP010	29	0	0	0	0	0	0	0	0	0	0	0	0	0	0	0	0
NP011	40	2	0	0	0	1	0	0	0	0	0	0	0	0	1	0	2
NP012	40	2	0	0	1	0	0	0	0	0	0	0	1	0	0	0	2
Total	478	25 (5,2%)	3 (5,1%)	3 (5,1%)	9 (15,5%)	10 (17,2%)	7 (12%)	2 (3,4%)	2 (3,4%)	7 (12%)	0	1 (1,7%)	4 (6,9%)	1 (1,7%)	6 (10,3%)	3 (5,1%)	---

The genomic topics outlined by Skirton (*n* 14) were analyzed to understand the extent of coverage of nursing programs and to this end the segments of text that referend to genomic topics were coded. We identified a total of 58 instances when genetic topics were included. Mendelian inheritance patterns were coded only three times across the nursing programs specifications. Descriptions of chromosomes, genes, and DNA, and their interrelationships were found nine times. Description of different causes of genetic disease were the most frequently covered topic, appearing ten times. Transcription of DNA to protein structure was covered seven times and the topic differences between inherited disorder and genetic susceptibility were identified six times. Description of different forms of testing and their applications were outlined three times and preventive actions or treatment referred four times.

However, communication of genetic information to families was addressed only twice and collection of a family history of a disease or condition was only found in one course. Furthermore, genetic risk assessment was included three times, and the psychosocial impact of genetic conditions was addressed twice, underscoring a lack of attention to these critical areas.

Despite the importance of addressing sources of relevant and reliable genetic information, none of the courses mentioned this aspect, and the profile and location of genetic services was referred only once. The ethical issues related to genetic status were coded seven times.

The content analysis based on the 14 genomic content topics across the 12 nursing programs (NP001 to NP012) demonstrated considerable variation in the number of topics covered. NP004 included the highest number, addressing nine of the 14 specific topics, followed by NP001, which also covered nine topics. In contrast, NP010 did not have any genomic content, since no specific topics were found, while NP002 and NP005 included only one topic. Programs such as NP006 and NP008 demonstrated moderate coverage, addressing seven and four topics, respectively. NP003, NP011 and NP012 address two genomic topics, and NP007 and NP009 three topics. This distribution reflects the uneven incorporation of genomic content among nursing programs, with only a few programs providing more comprehensive coverage, as shown in Table 4.

NP001 stands out for including a course entirely dedicated to genomics, where a broad range of genomic content was identified. However, this course is classified as an elective, meaning it is not mandatory and, therefore, not attended by all students enrolled in the program. This limits the exposure of the entire student cohort to critical genomic content, despite the breadth of topics covered in this course.

**Fig. 1:**
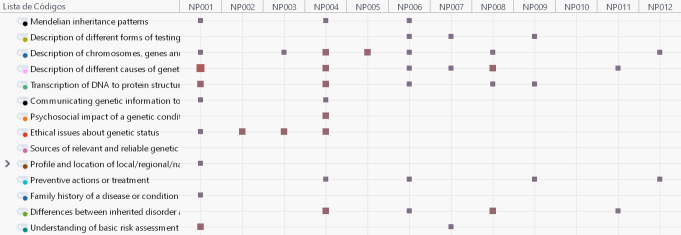
Codex matrix by nursing program

Figure 1 graphically represents the number of instances for each topic across the different nursing programs. The figure illustrates the distribution of instances, highlighting the variability in how genomic topics are addressed in each nursing program.

**Table 4:** Presence of specific genomic topics across nursing programs

Genomic content	Nursing Programs											
	NP001	NP002	NP003	NP004	NP005	NP006	NP007	NP008	NP009	NP010	NP011	NP012
Mendelian inheritance patterns	✓			✓		✓						
Description of different forms of testing and their applications						✓	✓		✓			
Description of chromosomes, genes and DNA and how they relate to each other	✓		✓	✓	✓	✓		✓				✓
Description of different causes of genetic disease	✓			✓		✓	✓	✓			✓	
Transcription of DNA to protein structure	✓			✓		✓		✓	✓			
Communicating genetic information to families	✓			✓								
Psychosocial impact of a genetic condition on the family				✓								
Ethical issues about genetic status	✓	✓	✓	✓								
Sources of relevant and reliable genetic information												
Profile and location of local/regional/nation genetic services	✓											
Preventive actions or treatment				✓		✓			✓			✓
Family history of a disease or condition	✓											
Differences between inherited disorder and genetic susceptibility				✓		✓		✓			✓	
Understanding of basic risk assessment	✓						✓					

Table 5 presents the distribution of the 25 courses that referred to genomic topics across different scientific areas. The majority of these courses (68%) were classified under Biomedical Sciences, indicating the dominant presence of genomic content in this area. Courses categorized under Ethics represented 16% of the total, while both Psychological and Social Sciences and Nursing Sciences accounted for 8% each. These data demonstrate that most courses incorporating genomic content belong to the biomedical field, with fewer courses addressing other scientific areas.

**Table 5:** Number and percentage of courses referred to genomic content by scientific areas

Total courses with genomic topics (*n* 25)		
Scientific areas	Total (n25)	Percentage
Biomedical sciences	17	68%
Psychological and Social sciences	2	8%
Ethics	4	16%
Nursing sciences	2	8%

## Discussion

The analysis of the genomic content across the 12 Portuguese nursing programs (NP001 to NP012) revealed substantial variability in the number of covered topics. Each program was assessed for the inclusion of 14 distinct genomic topics, with notable differences in the extent of coverage. NP004 emerged as the most comprehensive program, addressing nine of the 14 possible genomic topics. Similarly, NP001 also demonstrated a relatively high level of coverage, incorporating nine genomic topics. In contrast, programs such as NP002, NP003, and NP010 exhibited minimal inclusion, with NP010 notably lacking any genomic content. This variability underscores the inconsistency in the integration of genomics across nursing programs, highlighting significant disparities in the depth and range of genomic education provided. This lack of integration persists despite the well-established recognition in the literature of the critical role that genomics plays in nursing practice.

Similar results have been identified in nursing programs in the United States. However, despite a longer tradition of establishing competencies in genetics and genomics and greater recognition of their importance in nursing education, significant gaps in curricular integration still persist (Joffe et al. 2024). In a research study conducted by Joffe et al. (2024) that examined the integration of genomics in nursing programs utilizing an approach that include the analyses of publicly available course descriptions, only 5.6% of the programs include a fully dedicated genomic course, 50.9% of the programs have genomic content in the specifications of at least one course and the remaining 43.5% did not mention genomic content.

The genetic topics related to basic molecular biology and fundamental genetic principles tend to be more emphasized in nursing curricula compared to those focused on psychosocial aspects or patient-centered care. For instance, descriptions of chromosomes, genes, and DNA and the transcription of DNA to protein structure were coded more frequently. However, critical areas such as the communication of genetic information to families, psychological impact of a genetic condition, basic risk assessment and the collection of family history were significantly underrepresented. The distribution of genomic content across scientific areas reinforces the dominance of biomedical sciences in genomic content, with 68% of courses falling under this category. This is consistent with Joffe et al. (2024) findings, which also showed a strong emphasis on biomedical topics at the expense of psychosocial, ethical, and nursing-specific content. This suggests that nursing education places a stronger emphasis on the scientific foundations of genetics, perhaps reflecting a traditional biomedical approach. While a solid understanding of molecular genetics is essential, the limited attention to psychosocial and practical aspects highlights a crucial gap. Preparing nurses to apply genomic knowledge in clinical settings requires not only theoretical understanding but also the ability to provide holistic, patient-centered care, including counselling and ethical decision-making (Bayrak and Eren 2024; Calzone et al. 2024).

The literature highlights that gaps in genomics education contribute to persistently low levels of knowledge and confidence in genomic practice among both nursing students and clinical nurses (Ballad et al. 2024; Godino and Skirton 2012; McLaughlin et al. 2024; Seven et al. 2015). Some studies measure knowledge using The Genetic and Genomic in Nursing Practice Survey (GGNPS) developed by Calzone et al. (2014) and studies consistently report a low level of knowledge in genomics among both nursing students and practicing nurses (Ceylan et al. 2024; Chow et al. 2023; Wang et al. 2023; Yesilcinar et al. 2022). Other studies use the Genetic Nursing Concept Inventory (GNCI) developed by Ward et al. (Ward et al. 2016), and these studies also demonstrate low levels of knowledge in genomics (Adejumo et al. 2021; Dewell et al. 2020; Parviainen et al. 2023).

A recent study developed by Seed et al. (2024) shows that medical students generally have high confidence in basic genomics principles, however, this confidence drops significantly in clinical applications of genomics, with only 50% reporting a good understanding of the genetic contribution to diseases and just 28% feeling knowledgeable about clinically used genomic tests. The lack of confidence in these areas is attributed to gaps in knowledge, particularly in more complex topics. The study suggests that undergraduate curricula should be updated to better integrate genomic medicine (Seed et al. 2024). This includes enhancing the teaching of both basic science and clinical applications of genomics to address these knowledge gaps and ensure that future doctors are well-equipped to practice in an increasingly genomic healthcare environment (Seed et al. 2024). These findings align with our study, which also highlights gaps in the integration of genomic content within nursing curricula, emphasizing the need for a more comprehensive approach to preparing healthcare professionals for genomic healthcare.

Whitley et al. (2020) further elaborate on these challenges by emphasizing the critical importance of integrating genomics education into both foundational and advanced learning contexts. Their study highlights that equipping healthcare professionals and the public with the necessary knowledge to navigate the complexities of personal genomics is essential for addressing ethical, societal, and clinical challenges. Moreover, the authors stress the need for education that bridges the gap between basic genomic principles and their real-world applications, ensuring that professionals are prepared to leverage genomic advancements effectively in healthcare practice (Whitley et al. 2020).

In fact, if a nursing curriculum fails to encompass all relevant domains of genomics, it cannot be considered as fully competent in providing nurses with the comprehensive knowledge required for effective clinical practice. In this study, the maximum number of genomic topics identified within a nursing program was nine out of 14, indicating significant deficiencies in the breadth of genomic content offered.

These educational weaknesses are associated with difficulties in applying genomic knowledge effectively in clinical settings, which can compromise patient care and decision-making. Previous research has identified several strategies to address these educational gaps and enhance genomics education in nursing curricula. These strategies include the integration of genomics modules from the early stages of nursing education, the use of case-based learning and clinical simulations to apply genomic knowledge in real-world contexts, the involvement of genetic specialists in delivering lectures and hands-on workshops (Sharoff 2015; Tully et al. 2020; Zureigat et al. 2022) and even WhatsApp-based educational programs (Ceylan et al. 2024). Additionally, continuing education programs for practicing nurses are considered essential for keeping them up to date with the latest advancements in genomics, ensuring that they are better equipped to incorporate genomic knowledge into patient care and also in nursing research (Barbato et al. 2019). These strategies aim not only to improve theoretical understanding but also to build confidence and practical competence among nursing professionals in the application of genomics (Calzone et al. 2018a; Dewell et al. 2024).

Despite strategies to integrate genomics into nursing curricula, the research in genomic nursing field also identifies numerous barriers to the integration of genomics into nursing curricula. These barriers include insufficient resources, such as a lack of qualified faculty with expertise in genomics, and the limited time available within the already crowded nursing curriculum to adequately address complex genomic topics (Hines-Dowell et al. 2024; Parviainen et al. 2023). Additionally, many educators and healthcare professionals report low levels of confidence and knowledge in genomics, which can impede their ability to effectively teach the subject (Barbato et al. 2019; Mathis 2022; Read and Ward 2016; Smania et al. 2022). Furthermore, there is often no standardized approach to the core genomic content that should be included in nursing programs, leading to variability in the depth and breadth of genomics education across different institutions, as shown in this study.

These challenges underscore the need for systemic changes to overcome both structural and pedagogical obstacles, ensuring the comprehensive inclusion of a genomic knowledge matrix in nursing education and an early integration of genomic content at all levels of nursing education is crucial to ensure that future nurses recognize the importance of genomics in care delivery and research (Regan et al. 2019; Schluter 2023).

Our findings reveal significant gaps in the integration of genomic content within nursing programs, a concern that becomes more pressing when compared to the education provided to other healthcare professionals. While physicians often receive more comprehensive training in genomics, nursing education lags behind, limiting nurses’ ability to engage with genomic healthcare effectively. This disparity raises important questions about how genomics is being introduced in our country and underscores the urgent need for curricular reform in nursing education. Additionally, the growing role of genetic counselors, who are essential for interpreting genetic tests and guiding patients, highlights the need for interdisciplinary collaboration (Costa et al. 2024; Guimaraes et al. 2024; Paneque et al. 2022, 2023). In Portugal, a master’s degree in genetic counseling is already available, reflecting a recognition of the importance of specialized training in this field. This program includes courses such as *Genetic Counseling Principles and Techniques*, *Clinical Molecular Genetics* and *Bioethics and Genetics*, which provide extensive training in both clinical and psychosocial genomics. In contrast, nursing programs have not yet fully integrated such comprehensive genomic education. To ensure that nurses can work effectively alongside other healthcare professionals and contribute to genomic healthcare, it is crucial to revise nursing curricula to include both foundational genomics and its clinical applications. Such reform will better equip nurses to actively participate in interdisciplinary care teams and improve patient outcomes in an era increasingly driven by personalized healthcare. Interprofessional collaboration has the potential to improve patient care, reduce costs, and enhance the overall quality of service, particularly when integrating emerging fields like genomics into clinical practice (Ersig et al. 2025). Collaboration among healthcare professionals becomes a key component not only in clinical settings but also within educational programs that prepare healthcare workers (Costa et al. 2024). To foster effective collaboration, educational strategies could include joint curricula that allow students from different disciplines, such as nursing, genetic counselling, and medicine, to learn together (Clary-Muronda and Smith 2024). These curricula could focus on core genomic concepts and their application in diverse clinical scenarios, helping students to understand both the theoretical and practical aspects of genomics. Furthermore, interprofessional workshops or simulation exercises could be employed to provide students with hands-on experience working together in teams (Zureigat et al. 2022). These workshops would enable future healthcare providers to collaborate in real-time, simulating situations where genomic knowledge and interprofessional teamwork are required. Additionally, implementing these strategies throughout the education process will not only build a foundation of genomic knowledge across disciplines but also ensure that healthcare professionals are well-prepared to apply this knowledge in patient care. By promoting interprofessional collaboration both in education and clinical practice, we can create healthcare teams capable of delivering more integrated and personalized care (Limoges et al. 2024).

Although the importance of genomics in nursing practice is well recognized, there remains a lack of comprehensive research in Portugal, particularly regarding its integration into nursing curricula. National studies addressing these gaps are notably absent, which presents a significant barrier to advancing nursing education in this critical area. In the context of precision healthcare, it is imperative to invest in targeted research and curricular enhancements to equip nurses with the competencies needed for genomics-informed, patient-centered care. By fostering a robust understanding of both the theoretical and practical aspects of genomics, future nurses will be better prepared to meet the evolving demands of clinical practice and contribute effectively to interdisciplinary healthcare teams.

### Strengths and limitations

This study provides a valuable overview of the current state of genomic content integration in Portuguese nursing curricula, highlighting significant gaps that warrant attention. In the context of precision healthcare, these findings underscore the need for ongoing evaluation of how genomics is incorporated into nursing education to ensure that future nurses are adequately prepared to meet evolving clinical demands.

Several limitations of this study must be acknowledged. The analysis relied on publicly available course descriptions, which may not fully capture the depth or scope of the genomic content delivered in nursing programs. Detailed learning materials, instructional methods, and actual teaching practices were beyond the scope of this research, which limits the ability to draw conclusions about the extent of genomic education provided. Additionally, the study did not consider clinical learning environments where students might apply genomic knowledge, potentially take advantage of essential experiential learning opportunities, such as genetic counselling or family history collection. Future research could address these limitations by incorporating qualitative methods such as interviews with faculty and focus groups with curriculum developers. These approaches would provide deeper insights into the rationale behind curricular choices and the extent to which genomic content is intentionally integrated into nursing education. Additionally, further investigation is needed to explore the perspectives of nurses working in genetic healthcare settings regarding the additional education they feel would enhance their practice. Understanding these educational needs could inform the development of targeted educational strategies that bridge existing gaps in genomics nursing education. Moreover, the findings are specific to nursing programs in Portugal, which may limit their generalizability to other educational contexts.

### Recommendations

Further recommendations arise from the findings of this study. To address the identified gaps, nursing programs should prioritize the integration of comprehensive genomic content throughout the curriculum. This includes ensuring that core courses, particularly those in nursing practice, psychosocial care, and ethics, incorporate relevant genomic principles. Developing standardized guidelines for the inclusion of genomic content could help reduce variability across programs and ensure consistency in nursing education. Moreover, nursing regulatory bodies, higher education institutions, and professional organizations in Portugal could collaborate to establish policies supporting the integration of genomics into nursing curricula. One potential strategy could be the development of a genomic knowledge matrix that defines the minimum required genomic content for nursing programs, ensuring consistency across institutions. In addition, these bodies could collaborate to create accreditation standards that include genomic education as part of nursing program requirements.

Greater collaboration with genetic specialists and interdisciplinary teams can enhance the quality of education, providing nursing students with access to updated resources and expertise in genomics. In parallel with defining the genomic content that should be integrated into nursing curricula, it is equally important to establish and regulate the competencies that nurses must acquire in genomics. This process should leverage existing frameworks and guidelines developed in Europe, ensuring alignment with international standards and promoting consistency in the professional qualifications of nurses across different contexts. Nurses should also remain current with technological innovations in genomics, ensuring they are equipped to apply the latest advancements in clinical practice and deliver high-quality, genomics-informed care.

## Conclusions

This study highlighted significant variability, as well as the limited scope, of genomic content across a representative number of nursing programs in Portugal. While some programs include a broad range of topics, many focus predominantly on a biomedical approach, with minimal emphasis on psychosocial and patient-centered aspects of genomic care. Despite the growing recognition of genomics’ importance in nursing practice, the findings reveal substantial gaps in the integration of these topics, indicating the need for a more consistent and comprehensive approach to genomic education within nursing curricula. These findings are not exclusive to Portugal but reflect a broader global challenge in integrating genomics into nursing education. Studies from various countries have highlighted similar gaps in genomic literacy among nurses, emphasizing the need for comprehensive curricular reforms (Adejumo et al. 2021; Chow et al. 2023; Dewell et al. 2024; Joffe et al. 2024; Regan et al. 2019; Sharoff 2015; Whitley et al. 2020). The limited incorporation of genomics in nursing programs worldwide suggests a systemic issue that requires coordinated efforts at both national and international levels. Addressing this challenge through interdisciplinary collaboration, faculty development, and evidence-based educational strategies is essential to fully integrate genomics into nursing education (Limoges et al. 2024).

As precision medicine continues to evolve, it is essential that nursing programs adapt to include both theoretical knowledge and practical applications of genomics, ensuring that nurses are prepared to effectively integrate genomic information into clinical practice. Additionally, defining and regulating the competencies required for nurses in genomics, based on existing European frameworks, will help standardize and strengthen genomic education across programs.

This study underscores the urgency for curricular revision to address these gaps and ensures that future nurses are equipped with the knowledge and competencies necessary to deliver high-quality, genomics-informed care, meeting the demands of modern healthcare in the era of precision medicine.

## Data Availability

The data supporting the findings of this study are publicly available and consist of curricular programs which can be accessed through institutional websites.
